# Reply to Figueira *et al.*: Can NAD(P)^+^ transhydrogenase (NNT) mediate a physiologically meaningful increase in energy expenditure by mitochondria during H_2_O_2_ removal?

**DOI:** 10.1016/j.jbc.2021.100378

**Published:** 2021-03-15

**Authors:** Cody D. Smith, Cameron A. Schmidt, Kelsey H. Fisher-Wellman, P. Darrell Neufer

**Affiliations:** 1 East Carolina Diabetes and Obesity Institute, East Carolina University, Greenville, North Carolina, USA; 2 Department of Physiology, Brody School of Medicine, East Carolina University, Greenville, North Carolina, USA

In our recent publication (1), as well as an earlier publication from our lab (2), we demonstrate under specific experimental conditions designed to increase flux through redox buffering circuits in mitochondria, and thus NADPH demand, that at least a portion of the accompanying increase in *J*O_2_ (*i.e.*, proton conductance) is directly and reproducibly attributed to nicotinamide nucleotide transhydrogenase (NNT; *e.g.*, Fig. 3*D* in (1)). In their letter to the editor, Figueira *et al.* question whether flux through redox circuits linked to NNT can mediate a meaningful increase in energy expenditure. Their critique is based on two main points: 1) recent structural studies on NNT indicating a 1:1 stoichiometry of hydride transfer to NADPH generation, and 2) two prior studies that failed to show any difference in respiration in mitochondria with and without functional NNT (3, 4). Regarding the first point, we agree that recent structural studies of NNT (5), including a 2019 paper by Kampjut and Sazanov (6) (a citation we regrettably omitted), provide compelling evidence of an H^+^ to hydride transfer reaction stoichiometry of 1:1, which is considerably different than the apparent stoichiometry observed in our functional studies. We fully acknowledged this point in the discussion of our paper. However, NNT function cannot be determined solely from structural data. There are a number of plausible mechanisms that could explain a different apparent stoichiometry in respiring mitochondria, including a “permissible” transfer of protons by NNT under certain conditions, akin to the proton conductance of ANT that is independent of ATP/ADP exchange. On the technical side, it is also possible that *J*NADPH production was in excess of that required for H_2_O_2_ detoxification and/or that the rate of H_2_O_2_ emission as measured underestimated that produced. We attempted to control for the latter using inhibitors to both glutathione (BCNU) and thioredoxin reductase (auranofin), but such inhibitors have their own caveats, including slightly inhibiting respiration (as pointed out by Figueira *et al.* with respect to Fig. 4D). Regarding the apparent inconsistencies with the previous studies referenced, respiration was assessed in the study by Ronchi *et al.* (3) without accounting for potential differences in membrane potential. Identifying potential differences in proton conductance between experimental conditions requires comparing respiration at the same defined membrane potential. Parker *et al.* (4) did perform proton conductance assays in mitochondria with and without functional NNT, but not under conditions that would increase NNT activity (*i.e.*, elevate NADPH demand). While any increase in proton conductance back into the mitochondrial matrix does represent, by definition, an increase in energy expenditure, we agree with Figueira *et al.* that more research is needed to define the contribution of flux through NNT-linked redox circuits to energy expenditure *in vivo*.

## Conflict of interest

The authors declare that they have no conflicts of interest with the contents of this article.
